# Polysplenia Syndrome Complicated by Splenic Infarction: A Report of a Rare Case

**DOI:** 10.7759/cureus.38589

**Published:** 2023-05-05

**Authors:** Ruya A Abdullah, Mohammed A Alharbi, Faisal M Alnutaifi, Abdulrahman I Faqih, Khalid M Alduraibi, Ahlam S Alharbi

**Affiliations:** 1 Medicine, Ibn Sina National College, Jeddah, SAU; 2 General Practice, Umm Al-Qura University, Mecca, SAU; 3 General Practice, King Saud Bin Abdulaziz University for Health Sciences, Riyadh, SAU; 4 Family Medicine, Primary Health Care Center, Riyadh, SAU

**Keywords:** case report, computed tomography, congenital anomaly, splenic infarction, polysplenia syndrome

## Abstract

Polysplenia syndrome is a rare congenital anomaly characterized by multiple spleens and associated organ anomalies, which can present with serious complications such as splenic infarction. Diagnosis and management of the disorder can be challenging due to the presence of associated anomalies and the condition is often diagnosed incidentally. We report a case of a six-year-old girl with no significant medical history who presented to the emergency department with fever, abdominal pain, and vomiting. Physical examination and laboratory investigations showed leukocytosis, anemia, and elevated levels of C-reactive protein. A computed tomography scan revealed splenic infarction with polysplenia syndrome. The patient received intravenous antibiotics and pain management and was closely monitored for complications such as sepsis. Early diagnosis and appropriate management are essential to prevent complications, and close monitoring and follow-up are necessary for long-term management.

## Introduction

Polysplenia syndrome is a rare congenital anomaly characterized by multiple spleens and various associated organ anomalies. The presence of multiple small spleens and associated congenital anomalies, such as cardiovascular defects and gastrointestinal malformations, can make diagnosis and management of the condition particularly challenging [1]. It affects approximately one in 10,000 live births. The disorder is often diagnosed incidentally during imaging studies for unrelated medical conditions or when patients present with symptoms of associated anomalies (e.g., abdominal pain) [1]. However, polysplenia syndrome may present with splenic complications, such as splenic infarction, as in the case presented here.

Splenic infarction is a rare but serious complication of polysplenia syndrome. It occurs due to the occlusion of the splenic artery or its branches, leading to a lack of blood flow and subsequent ischemia and necrosis of the splenic tissue [2]. Patients with polysplenia syndrome are at increased risk of splenic infarction, as they have abnormal vasculature and an increased number of accessory spleens [1,2].

## Case presentation

A six-year-old girl presented to the emergency department with complaints of fever, abdominal pain, and vomiting for the past two days. The patient had no significant medical history or known allergies, and there was no family history of similar symptoms. During the physical examination, the patient appeared ill, and her temperature was 38.8°C. Abdominal examination revealed tenderness in the left upper quadrant with no palpable masses. There was no evidence of jaundice or lymphadenopathy. The cardiovascular and respiratory examinations were normal.

Laboratory investigations showed leukocytosis with a white blood cell count of 16.5x10^9^/L, a hematocrit of 34%, and elevated levels of C-reactive protein at 140 mg/L. The platelet count was within normal limits. The liver function tests and electrolyte panel were also normal.

**Figure 1:**
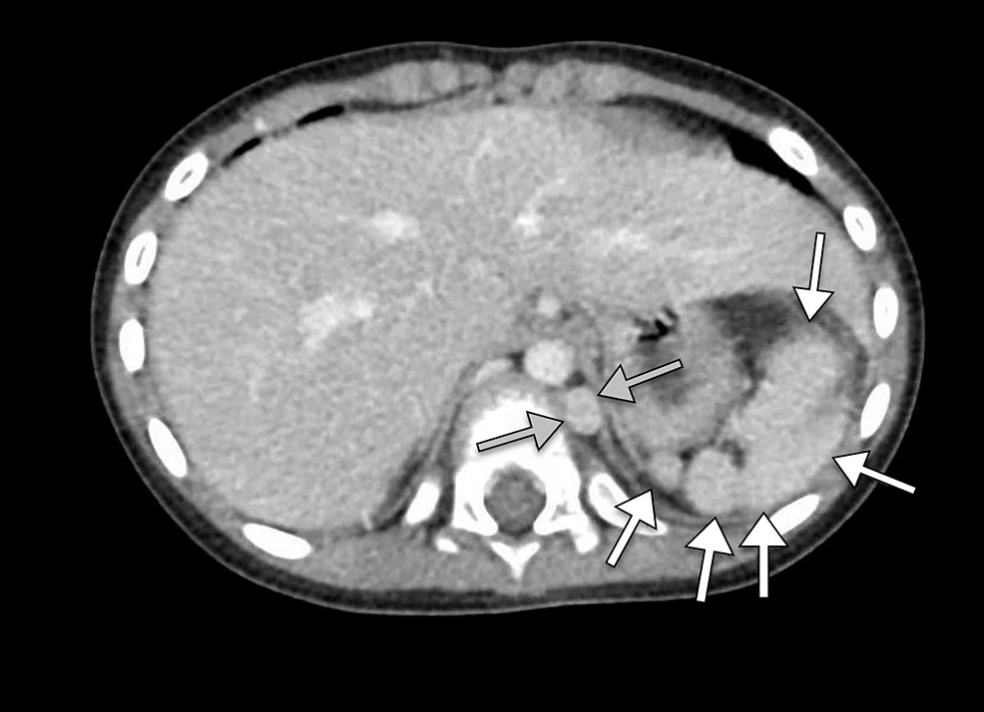
Axial CT image of the abdomen demonstrating multiple spleens (white arrows) and a left-sided inferior vena cava (gray arrows). CT, computed tomography.

**Figure 2:**
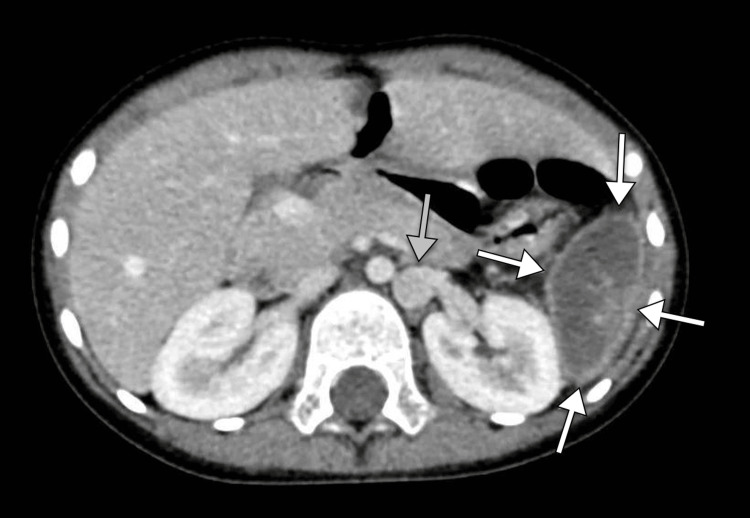
Axial CT image of the abdomen displaying a homogeneous hypodense mass with an enhancing wall (white arrows) and a left-sided inferior vena cava (gray arrows). CT, computed tomography.

**Figure 3:**
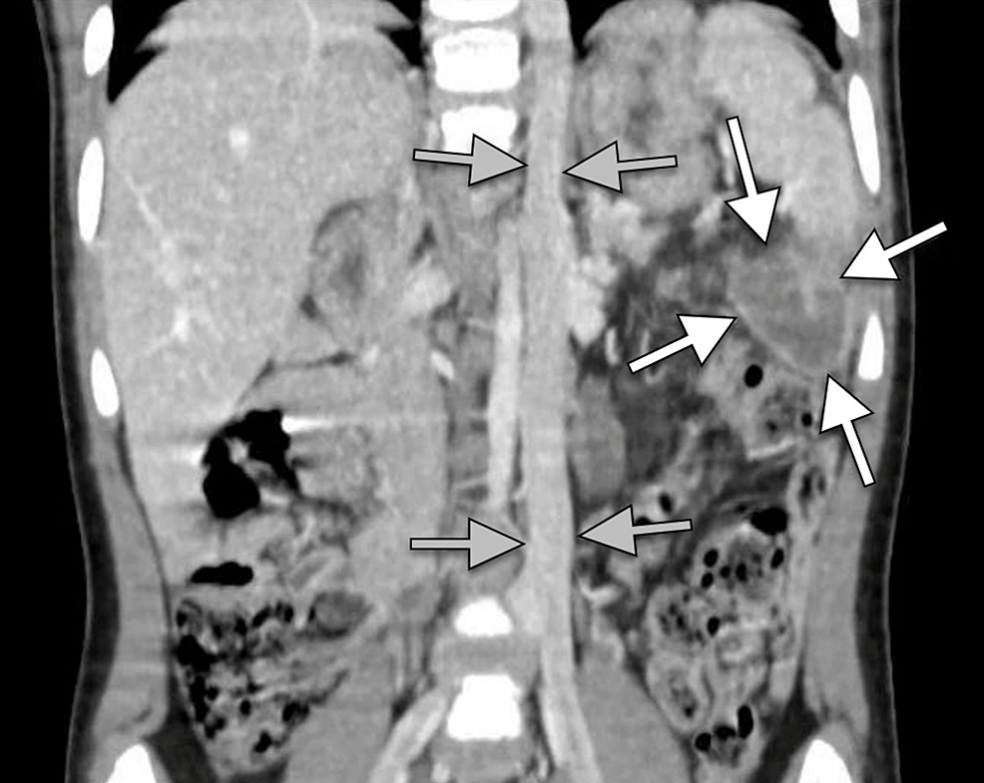
Coronal CT image of the abdomen presenting a homogeneous hypodense mass with an enhancing wall (white arrows) and the continuation of the hemiazygos vein as a left-sided inferior vena cava (gray arrows). CT, computed tomography.

The bedside ultrasound examination of the abdomen was of limited diagnostic value due to excessive intra-abdominal gas. Hence, a computed tomography scan of the abdomen revealed multiple spleens on the left side, with a homogeneous hypodense mass showing enhancement of the capsule and non-enhancement of the parenchyma, indicating splenic infarction. The scan also showed a short pancreas and interruption of the inferior vena cava with hemiazygos continuation of the left-sided inferior vena cava. Such findings were consistent with splenic infarction with polysplenia syndrome (Figures 1-3).

The patient received intravenous antibiotics with ceftriaxone for possible sepsis and was given pain management with acetaminophen and ibuprofen as needed. Repeat laboratory investigations on day 2 showed a reduction in leukocytosis to 10.2x10^9^/L and a decrease in C-reactive protein to 80 mg/L. The patient was closely monitored for complications such as sepsis.

A hematologist was consulted for further management, and additional laboratory investigations, including a blood culture, were obtained, which came back negative for bacterial growth.

During the hospital stay, the patient’s pain was well controlled with acetaminophen and ibuprofen as needed. She also received intravenous fluids to maintain hydration and electrolyte balance. As the patient’s leukocytosis and C-reactive protein levels decreased, the antibiotics were discontinued on day 5. After a 10-day hospital stay, the patient was discharged with a follow-up specialist consultation. The patient’s parents were advised to monitor the patient for signs of infection, such as fever and worsening abdominal pain, and to seek medical attention if such symptoms develop.

## Discussion

Polysplenia syndrome is a complex disorder that poses a diagnostic challenge due to its wide-ranging clinical presentation. The diagnosis is often made incidentally in imaging studies, as in this case. The presence of multiple spleens makes it difficult to distinguish between normal and abnormal anatomy, which can complicate the diagnosis of other associated anomalies. The typical presentation of polysplenia syndrome is variable, and patients may be asymptomatic or present with a range of symptoms, such as abdominal pain, as observed in this case [2].

Splenic infarction is an uncommon but well-recognized complication of polysplenia syndrome [3]. The symptoms of splenic infarction can mimic other conditions such as acute appendicitis, pneumonia, or urinary tract infection, making it a challenging diagnosis [2]. In this case, the patient presented with fever, abdominal pain, and vomiting, which are common symptoms of splenic infarction. The diagnosis was confirmed by imaging studies, which showed a homogeneous hypodense mass with the enhancement of the capsule and non-enhancement of the parenchyma, indicating splenic infarction.

The management of splenic infarction in polysplenia syndrome is mainly supportive, with antibiotics and pain management as the primary treatments [2,4]. The goals of therapy are to prevent complications such as sepsis, maintain hydration and electrolyte balance, and manage pain [3]. In this case, the patient was started on intravenous antibiotics and received pain management with acetaminophen and ibuprofen. As the patient’s leukocytosis and C-reactive protein levels decreased, the antibiotics were discontinued, and the patient was monitored for signs of infection [2].

The prognosis for patients with polysplenia syndrome and splenic infarction is generally favorable, with most patients recovering without complications [3]. However, there is a risk of recurrent splenic infarction, which may require splenectomy, especially if there is a significant decrease in spleen function [5]. In this case, the patient received a follow-up specialist consultation to monitor her condition and ensure appropriate long-term management.

## Conclusions

In conclusion, this case presents a rare congenital anomaly of polysplenia syndrome, complicated by splenic infarction. The diagnosis of polysplenia syndrome is challenging due to the variable presentation and associated anomalies. In this case, imaging studies were crucial in confirming the diagnosis of splenic infarction. Early diagnosis and appropriate management are essential to prevent complications such as sepsis and ensure favorable outcomes. Close monitoring and appropriate follow-up are also necessary to prevent recurrent infarction and ensure appropriate long-term management.
